# Acute and Chronic Physical Activity Increases Creative Ideation Performance: A Systematic Review and Multilevel Meta-analysis

**DOI:** 10.1186/s40798-022-00444-9

**Published:** 2022-05-06

**Authors:** Christian Rominger, Martha Schneider, Andreas Fink, Ulrich S. Tran, Corinna M. Perchtold-Stefan, Andreas R. Schwerdtfeger

**Affiliations:** 1 Institute of Psychology, University of Graz, Graz, Austria; 2 Department of Cognition, Emotion, and Methods in Psychology, School of Psychology, University of Vienna, Vienna, Austria

**Keywords:** Divergent thinking, Creative potential, Bodily movement, Open problem solving

## Abstract

**Background:**

Physical activity is a health-relevant lifestyle factor associated with various benefits on physical and mental health. Several meta-analyses indicated effects of acute and chronic physical activities on elementary cognitive functions such as executive control processes, memory, and attention. Meta-analytic evidence on the effects of physical activity on creative idea generation, which involves a conglomerate of these elementary cognitive functions, is largely missing.

**Objective:**

A twofold approach was used to evaluate (1) if there is an association between habitual physical activity and creative ideation and (2) if physical activity interventions (acute and chronic) enhance creative ideation performance.

**Methods:**

Multilevel meta-analytic methods were applied to (1) evaluate the cross-sectional association between creative ideation performance and measures of habitual physical activity and (2) the effect of physical activity on creative ideation performance. Indicators of creative ideation (fluency, flexibility, originality, elaboration, or composite score), creativity domain (verbal, figural), population (adults, children), gender, study quality, and publication year served as moderator variables for both meta-analyses. Analyses of intervention studies additionally examined the moderator variables study design (between, within), time of measurement (during, after), and implementation of intervention (acute, chronic).

**Results:**

The applied meta-analytic multilevel analysis indicated a medium effect for cross-sectional studies (*r* = 0.22, SE = 0.06, *p* = 0.002, 95% CI [0.10–0.34]) based on 17 effects sizes from seven studies. The pooled effects of 28 intervention studies, providing 115 effect sizes, indicated a medium effect size of Hedges’ *g* = 0.47 (SE = 0.09, *p* < 0.001, 95% CI [0.30–0.65]). Furthermore, a stronger effect was observed for chronic interventions of several days or weeks in comparison with acute interventions of one single bout.

**Conclusion:**

This study adds important new meta-analytic evidence on the beneficial role of physical activity beyond mental and physical health outcomes: Physical activity has a positive impact on creative ideation, which expands the literature on the role of physical activity in more elementary cognitive functions such as executive control, memory, and attention. Moderator analyses suggested that chronic interventions showed stronger effects than single bouts of physical activity. Rigorously conducted randomized controlled intervention studies and more cross-sectional studies are needed to broaden the evidence in this nascent field of research.

**Supplementary Information:**

The online version contains supplementary material available at 10.1186/s40798-022-00444-9.

## Key Points

- Cross-sectional studies and interventional studies show a medium-sized positive effect of physical activity on creative ideation performance.
- The effects of chronical physical activity are stronger compared to the effects of acute physical activity.
- Rigorously conducted randomized controlled intervention studies and more cross-sectional studies are needed to broaden the evidence.

## Introduction

Physical activity facilitates (mental) health and increases cognitive functions such as executive control, attention, and memory processes [1–7]. The dynamic interplay of specific cognitive functions is essential to produce creative solutions that are surprising, original, and applicable [8]. While research on creativity has long been restricted to classic domains such as music, science, or the arts, recent studies have begun to discover the prominent role of creativity in the domain of sports. For instance, creative cognition and cognitive flexibility can facilitate game-related decision-making and prevent sport injuries [9]. Furthermore, creative cognition is an important factor in team sports with the potential to determine victory or defeat [10]. Although the impact of physical activity on cognitive functions is well known, reviews and meta-analyses have largely ignored its potentially enhancing effect on creative idea generation.

Can physical activity stop mental blocks and increase the uniqueness of ideas [8, 11–13]? And if so, can even a short run boost creative thoughts? Or is it necessary to train for weeks to finally achieve a higher creative output? Are different domains of creative ideation affected to the same extent? Is the effect of physical activity restricted to quantitative facets of creativity (e.g., number of generated ideas), or does it similarly apply to creative quality (e.g., originality)? Although an increasing number of empirical studies have targeted these exciting questions, not all of them have confirmed a positive link between physical activity and participants’ creative potential (i.e., performance in a divergent thinking task [14]; for a critical review see [15]). The increasing number of studies and inconsistent results call for a meta-analysis, which quantifies the overall effect of physical activity on creative ideation and examine relevant moderators. But first, why should a walk, a run, or a higher fitness level boost creativity at all?

Importantly, creative ideation can be considered the result of a complex interplay of cognitive functions, which is outlined in prominent dual-process models of creative thinking [16, 17]. Specifically, dual-process models suggest a dynamic interplay of associative (divergent) and executive (convergent) processes that establish generative and elaborative/evaluative modes of creative thinking. Furthermore, creative ideation performance is associated with higher executive control [18–22] and fluid as well as crystallized intelligence [23]. In line with these behavioral findings, neuroscientific creativity studies indicated higher involvement of brain areas associated with executive control [24–26] (but see [27]; for meta-analyses see [28–30]), memory processes [31, 32], and internal attention [33] during creative idea generation tasks. Taken together, physical activity may increase creative ideation performance via improving executive control, attention processes, and memory [34, 35].

The enhancement of cognitive functioning through physical activity is a field of increased interest [6]. Etnier and colleagues [2] conducted one of the first meta-analyses on cognitive functioning and physical activity, which showed a small positive overall effect (*d* = 0.25). Chang et al. [36] indicated a positive effect of acute (i.e., one single bout of) exercise on information processing, attention, crystallized intelligence, and executive functions [37–39]. Ludyga et al. [40] confirmed these findings and suggested that age is an important moderator, as the effects of physical activity were strongest among preadolescent children and older adults. Wilke et al. [9] extended this finding to acute effects of resistance training (i.e., strength training involving series of different exercises in the upper and lower limbs), which also enhances cognitive functioning in healthy adults. Furthermore, the meta-analysis of Etnier et al. [34] indicated that not only single bouts of exercises, but also chronic (i.e., longer lasting and repeated) physical activities have a positive pooled effect on cognitive performance [6, 7]. This indicates that a broad range of physical activities is associated with a variety of cognitive function increases. However, although the impact of acute and chronic physical activity on basic cognitive functions has been replicated in several meta-analyses [9, 34, 35, 41, 42], it is still unclear if physical activity enhances creative ideation performance.

While some empirical studies indicated an association between creative ideation performance and acute as well as chronic physical activity [43, 44], others did not [45]. To the best of our knowledge, meta-analytic approaches on this topic are very rare [15, 46]. Only Chang et al. [36] evaluated performance changes in divergent thinking tasks (i.e., alternate uses tasks) after acute physical activity. They found a weakly positive, but not significant pooled effect size of *d* = 0.11. Yet, this analysis included only 26 effects and was not specifically designed to investigate creative ideation performance. Hence, systematic quantitative aggregation of studies examining associations between physical activity and creative ideation is needed.

Of note, creativity research on the boosting effects of physical activity is rather diverse, and studies largely differ in design (e.g., interventional, cross-sectional), type of physical activity (e.g., aerobic exercise, running), implementation of intervention (e.g., acute, chronic), time of measurement (e.g., during, after an intervention), and the creativity domain of the task (e.g., verbal, figural). Furthermore, the extant literature differentiates between qualitative and quantitative indicators of creative ideation performance [23]. In particular, the number of different ideas (i.e., fluency) is the most frequently applied quantitative measure of creative thinking, besides flexibility (i.e., number of different categories used to produce ideas) and elaboration (i.e., number of details added to an idea). Qualitative indicators, on the other hand, are originality and other measures of creativity, which are traditionally assessed with frequency-based scores and external creativity ratings [47, 48]. Furthermore, some studies utilized composite scores of creativity (e.g., adding up the originality of all responses), thus aggregating qualitative and quantitative aspects [49].

Using multiple creativity indices from single studies violates the criterion of effect size independence in meta-analysis, which is why this study applied a multilevel meta-analytic approach with three levels [50–52]. We performed two separate analyses. First, we focused on cross-sectional data, which allowed analysis of the association between habitual physical activity (both self-reported and behavioral) and creative ideation performance. Second, we meta-analyzed intervention studies, which experimentally modified physical activity to increase creative ideation performance. This two-step approach allowed us to investigate, first, if an association between habitual physical activity and creative ideation performance exists [43] and, second, whether physical activity may have causal effects on creative ideation performance outcomes [6, 53].

## Methods

### Search Strategy

A systematic literature search for studies examining physical activity and creative ideation performance was carried out, based on the guidelines of the PRISMA statement [54]. Relevant articles were identified in the following online databases: Scopus, PsyArXiv, Google Scholar, Zenodo, PsycINFO, PubMed, Web of Science, CINAHL, and ProQuest. The search strings comprised terms describing physical activity (physical activity, bodily movement, aerobic exercise, exercise program, dancing, physical fitness, walking, and running) and terms describing creative ideation performance (creativity, creative thinking, divergent thinking, creative potential, open problem-solving, creative ideation, originality, and fluency; see Additional file 1 for the exact search strings for each database). The date of last search was January 2022. Review articles, qualitative research, case studies, and study protocols were excluded. Furthermore, the reference sections of relevant articles were screened for additional research [15, 36, 46]. There were no time or language restrictions. Both published and unpublished studies (e.g., dissertations), cross-sectional, and longitudinal/intervention studies were eligible for analysis.

### Inclusion and Exclusion Criteria

Studies were required to report measures of creative ideation performance. For the purpose of this study, we focused on the (more common) divergent thinking tasks of creativity [55] and the relevant indicators of quantity and quality of ideas. Performance data on convergent creativity tasks, such as the remote association task [56], were not extracted (similar approach, see [23].) Physical activity was defined as any kind of (bodily) movement (produced by skeletal muscles) that results in a substantial increase over the resting energy expenditure [39, 57]. Studies investigating movements of lower energy expenditure, such as hand waving [58] and hand contractions [59, 60], were thus excluded from analysis. Eligible studies comprised different types of physical activity such as running and aerobic exercise. For the meta-analysis of cross-sectional data, studies with various measures of physical activity, such as self-report, fitness-test, and accelerometers, were eligible.

### Data Extraction and Coding

Extracted data included (a) basic study information (authors, publication year, mean age), (b) indicators of creative ideation (fluency, flexibility, originality, elaboration, and composite score), (c) creativity domain (verbal, figural), (d) population (adults, children), (e) gender (percentage of female participants), (f) implementation of intervention (acute, chronic), (g) time of measurement (during, after), (h) study design (between, within), (i) effect size measures (effect size, standard error, standard deviation), and (j) study quality (study quality score, see Additional file 2). Study authors were contacted in cases of insufficient data for effect size calculation.

### Study Quality and Risk of Bias

We applied two scales adapted from Frith et al. [15] to assess the study quality of cross-sectional and intervention studies. The protocols comprised six items (for cross-sectional studies) and eight items (for intervention studies), focusing on bias detection, control group/condition, randomization, and creative ideation task specifications (Additional file 2). Each item was scored 1 if the respective criterion was met and 0 if not. The resulting study quality score served as moderator in analysis. For intervention studies, the maximum of the quality score was 7 and the lowest score was 2, with higher scores indicating higher quality of the study (see Table 2; for cross-sectional studies: Max = 5, Min = 3; see Table 1). Studies were not excluded based on quality.

### Statistical Analysis

Most studies reported effect sizes for more than one indicator of creative ideation performance (e.g., fluency, originality). The independence of effect sizes is crucial to avoid an overlap of information of the included effect sizes and to provide unbiased effect size estimations [50, 52]. The present multilevel meta-analysis structure integrated multiple effect sizes both within and between studies by distinguishing three levels of variance. The sampling variance of the extracted effect sizes (i.e., outcomes) was modeled at level 1, the variance of effect sizes within the same study at level 2, and the variance of effect sizes between different studies at level 3 [50, 52]. Heterogeneity was examined with moderator analysis if less than 75% of the total amount of variance could be attributed to the sampling variance at level 1 [61], which indicated that a meaningful amount of variance lay within and between the studies (i.e., at level 2 and level 3 [52]).

Two separate multilevel meta-analyses were conducted. The first meta-analysis focused on cross-sectional effects of physical activity on creative ideation performance and used the correlation coefficient *r* as the effect size measure. Quantification of the effect size magnitude was based on the thresholds devised by Cohen: small (0.1), medium (0.3), and large (0.5) [62]. A positive effect size indicated that higher levels of habitual physical activity were associated with a higher level of creative ideation performance. The second meta-analysis examined the effects of physical activity interventions on creative ideation. This analysis used Hedges’ *g*, which provides a better effect size estimation for small sample sizes as compared to Cohen's *d* [62]. Quantification of the effect size magnitude for Hedges’ *g* was performed using the thresholds defined for Cohen’s *d* (see above; [62]). A positive effect size indicated higher levels of creative ideation after the physical activity intervention than before. The meta-analysis of intervention data included studies with between- and within-subjects designs. The correlation between two time points of assessment was set to *r* = 0.50 to estimate the corresponding effect sizes for studies with a within-design not reporting this association. We further calculated sensitivity analyses by applying the correlation coefficients of *r* = 0.10 and *r* = 0.90.

Statistical analysis was performed with *R* [63], using the rma.mv function of the metafor package ([64]; for a detailed tutorial, see [52]). Restricted maximum likelihood (REML) estimation method was used for estimating parameters of the meta-analytic model [50, 52]. Moderator analyses for categorical and continuous moderator variables were conducted (e.g., study quality). Dummy-coded variables were created for the categorical moderators, comprising indicators of creative ideation performance (flexibility, originality, elaboration, or composite score vs. fluency [reference category]; for a similar approach, see [23, 65]), population (adults vs. children [reference category]), and creativity domain (verbal vs. figural [reference category]). For the meta-analysis of intervention studies, the implementation of intervention (chronic vs. acute [reference category]), time of measurement (during vs. after [reference category]), and the study design (between vs. within [reference category]) were added as further categorical moderators. For both meta-analyses, publication year, gender (percentage of female participants), and study quality were treated as continuous moderators.

We provide visualization of individual studies’ results using forest plots for meta-analyses with multiple outcomes [66]. Before drawing the forest plot, separate random-effects meta-analyses were conducted for each study. Publication bias was examined through funnel plots [66] and Egger’s regression test. Publication bias refers to the problem that studies with significant results, and large effect sizes are more likely to be published than studies without significance [62]. This can lead to a bias in effect size estimation.

**Table 1:** Study characteristics: cross-sectional studies

Study	Year	Participants	Age (mean, SD/range)	Creative ideation task	Physical activity measure	Study quality
Cantarero and Carranque [67]	2016	*n* = 40	32.95 (9.07)	Creative imagination test for adults [68]	International physical activity questionnaire [69]	3
Cavallera et al. [70]	2011	*n* = 61	21.64 (2.85)	Torrance test of creative thinking [71]	Hours of sport activity per week	4
Chen et al. [72]	2021	*n* = 40	22.98 (1.95)	Alternate uses test [73]	International physical activity questionnaire [69]	4
Latorre Román et al. [74]	2017	*n* = 308	9.72 (1.25)	Creative imagination test for children [75]	Fitness-test battery (20 m running speed)	4
Perchtold-Stefan et al. [76]	2020	*n* = 98	23.06 (3.40)	Verbal imagination subscales of Berliner Intelligenzstruktur-test [77] test for creative thinking-drawing production [78]	Freiburger questionnaire on physical activity [79]	4
Piya-Amornphan et al. (1–3) [80]	2020	*n*_1_ = 521 *n*_2_ = 487 *n*_3_ = 439	6–9 1–13 14–17	Test for creative thinking-drawing production [78]	Thailand physical activity children survey-the student Questionnaire [81]	3
Rominger et al. [43]	2020	*n* = 79	22.95 (3.34)	Alternate use task [73] Torrance test of creative thinking [71]	Tri-axial acceleration sensors (counts/min)	5

*n* number of participants, *n*_*i*_ number of participants in the independent subsample *i*, numbers in parentheses designate the independent subsamples *i*, e.g., Piya-Amornphan et al. (1–3)

## Results

### Study Selection and Study Characteristics

**Fig. 1:**
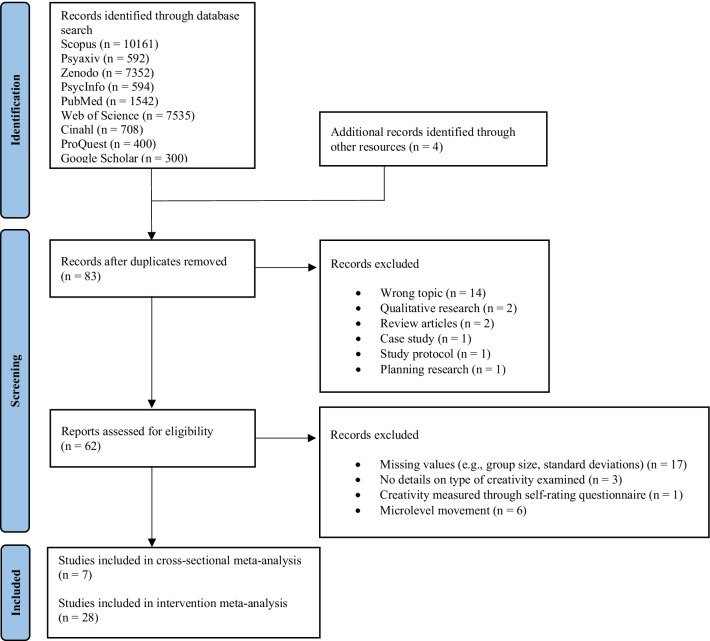
PRISMA flow diagram

Database search identified a total of 29,184 articles. Four articles were identified through other resources. After removing duplicates and screening titles and abstracts, 83 articles remained eligible for further examination. In total, another 48 articles had to be excluded that presented either another topic (14), qualitative research (2), or planned research (1), were a review article (2), a case study (1), or a study protocol (1); 17 studies did not provide sufficient data to calculate effect sizes, 6 studies investigated movements with low energy expenditure, 1 study measured creativity using a self-description scale, and 3 studies provided no details on the type of examined creativity. A total of 35 studies were included in the analyses (i.e., 7 cross-sectional studies and 28 intervention studies; see Fig. 1). Studies were published between 1985 and 2022. For detailed study description, see Table 1 and Table 2.

**Table 2:** Study characteristics: Intervention studies

Study	Year	Participants	Age (mean, SD/range)	Design	Physical activity intervention	Control	Creative ideation task	Study quality
Aga et al. [82]	2021	*n* = 40 Exercise group: *n* = 20 Control group: *n* = 20	22.98 (1.95)	Between-subject	Cycling (15 min)	Reading (15 min)	Alternate uses test [73]	7
Blanchette et al. [83]	2005	*n* = 60	20 ( −)	Within-subject	Aerobic exercise (30 min)	No exercise (30 min)	Torrance test of creative thinking [71]	4
Bollimbala et al. [84]	2019	*n* = 34 Experimental group: *n* = 17 Control group: *n* = 17	12 (1.29)	Between-subject (Parallel group)	Dance intervention (20 min)	Sitting (20 min)	Alternate uses test [73]	6
Bollimbala et al. [85]	2020	*n* = 92 Experimental group: *n* = 46 Control group: *n* = 46	22.04 (0.84)	Between-subject (Parallel group)	Yoga (20 min)	Case study (20 min)	Alternate uses test [73]	7
Bollimbala et al. [86]	2021	*n* = 68 Dancing group: *n* = 34 Control group: *n* = 34	21.6 (0.88)	Between-subject	Dancing (15 min)	Socializing	Alternate uses test [73]	5
Campion and Levita [87]	2014	*n* = 56 Dancing group: *n* = 15 Cycling group: *n* = 14 Music group: *n* = 14 No exercise group: *n* = 13	20.4 (1.31)	Between-subject	Cycling, dancing (5 min)	Listening to music, sitting quietly (5 min)	Torrance test of creative thinking [71]	5
Donnegan et al. (1–2) [88]	2018	Yoga group: *n*_1_ = 19 Aerobic group: *n*_2_ = 18	Yoga: 41.3 (−) Aerobics: 48.4 (−)	Within-subject	Yoga (75 min), aerobic (45 min)	–	The abbreviated Torrance test for adults [89]	3
Frith and Loprinzi [45]	2018	*n* = 32	23.1 (3.39)	Within-subject	Treadmill walking (15 min)	Sitting (15 min)	Alternate uses task [73]	7
Frith et al. [90]	2021	*n* = 32	22.66 (3.23)	Within-subject	Treadmill walking (20 min)	Sitting (20 min)	Instances creativity task [91]	7
Gondola [92]	1986	*n* = 19	9.77 (1.11)	Within-subject	Running (20 min/twice a week/six weeks)	–	Alternate uses test [73]	3
Herman-Tofler et al. [93]	1998	*n* = 51 Aerobic exercise group: *n* = 26 Physical education group: *n* = 25	–	Between-subject (Parallel group)	Aerobics (25 min/ three times a week/ eight weeks)	Physical education (25 min/ three times a week/eight weeks)	Torrance test of creative thinking [71]	3
Latorre Román et al. [94]	2018	*n* = 96 Aerobic game group: *n* = 48 Control group: *n* = 48	9.84 (1.12)	Between-subject (Parallel group)	Aerobic game session (45 min)	No exercise	Prueba de imaginacion creativa-Ninos [75]	6
Latorre Román et al. [95]	2021	*n* = 114 Active recess program group: *n* = *58* Control group: *n* = *56*	Experimental: 9.76 (1.09) Control: 9.77 (1.11)	Within-subject	Active recess program (about 20 min/three times a week/ 10 weeks)	–	Prueba de imaginacion creativa-Ninos [75]	5
Leung et al. [96]	2012	*n* = 104	–	Between-subject (Parallel group)	Free walking (2 min), rectangular walking (2 min)	Sitting (2 min)	Droodle and Lego task	4
Ludyga et al. [97]	2020	*n* = 34	12.8 (1.8)	Crossover	Aerobic exercise (20 min)	–	Alternate uses task [73]	6
Main et al. [98]	2020	*n* = 29	20.4	Mixed measure (2 × 2)	Treadmill walking (8 min/ 4 × 2 min)	Sitting (8 min/ 4 × 2 min)	Alternate uses task [73]	5
Matsumoto et al. [99]	2022	*n* = 22	21.36 (1.33)	Within-subject	Stair-climbing	–	Alternate uses test [73]	7
Murali and Händel (1–3) [100]	2022	*n*_*1*_ = 20 *n*_*2*_ = 17 *n*_*3*_ = 23	18–35 18–35 18–35	Within-subject	Walking	Sitting	Alternate uses task [73]	7
Netz et al. [101]	2007	*n* = 58 Moderately-intense aerobic group: *n* = 20 Moderate aerobic group: *n* = 20 Control group: *n* = 18	54.99 (3.14) 56.12 (3.4) 54.75 (3.01)	Between-subject (Parallel group)	Aerobic training (until participants reached their limit)	Watching a movie	Alternate uses test [73]	7
Oppezzo and Schwartz (1–3) [44]	2014	*n*_1_ = 48 *n*_2_ = 16 *n*_3_ = 10	–	Within-subject	Walking (treadmill and outside)	Sitting	Alternate uses test [73]	5
Österberg and Olsson [102]	2021	*n* = 51 Dancing group: *n* = 25 Control group: *n* = 26	12 ( −)	Between-subject	Dancing (3 min)	No exercise	Adopted idea generation test from Finke et al. [103]	2
Patterson et al. [104]	2018	*n* = 20	21.35 (1.3)	Within-subject	Treadmill walking (15 min)	–	Alternate uses task [73]	4
Richard et al. [105]	2021	*n* = 66 Aerobic dance group: *n* = 30 Control group: *n* = 36	23.56 (2.66)	Between-subject	Aerobic dancing (30 min/ twice a week/five weeks)	Received 3 short readings about creativity	Runco creative assessment battery	7
Ruiz-Ariza et al. [106]	2019	*n* = 184 Interval training group: *n* = 90 Static stretching group: *n* = 94	13.73 (1.34)	Between-subject (Parallel group)	High-intensity interval training (16 min/ twice a week/ 12 weeks)	Static stretching (16 min/ twice a week/ 12 weeks)	CREA test [107]	4
Steinberg et al. [108]	1997	*n* = 63	19–59	Within-subject	Aerobic workout (17 min) and dance (17 min)	Watched a documentary of similar duration	Torrance’s unusual uses test of creative thinking [71]	3
Tilp et al. [109]	2020	*n* = 35 Experimental group: *n* = 17 Control group: *n* = 18	12.26 (0.78)	Within-subject	Motor-coordinative exercise intervention (30 min/five times a week/four weeks)	Waiting for intervention	Alternate uses task [73]	5
Young-Mi and Hye-Jeon [110]	2016	*n* = 30 Dance art group: *n* = 15 Science class group: *n* = 15	–	Between-subject (Parallel group)	Dance art group (180 min/once a week/15 weeks)	Science experiment class (180 min/once a week/15 weeks)	Torrance test of creative thinking [71]	4
Zhou et al. [111]	2017	*n* = 63	21.25 ( −)	Within-subject	Walking	Standing in the center of the room	Consequences imagination task	6

*n* number of participants, *n*_*i*_ number of participants in the independent subsample *i*, numbers in parentheses designate the independent subsamples *i*, e.g., Donnegan et al. (1–2)

### Cross-Sectional Studies

**Fig. 2:**
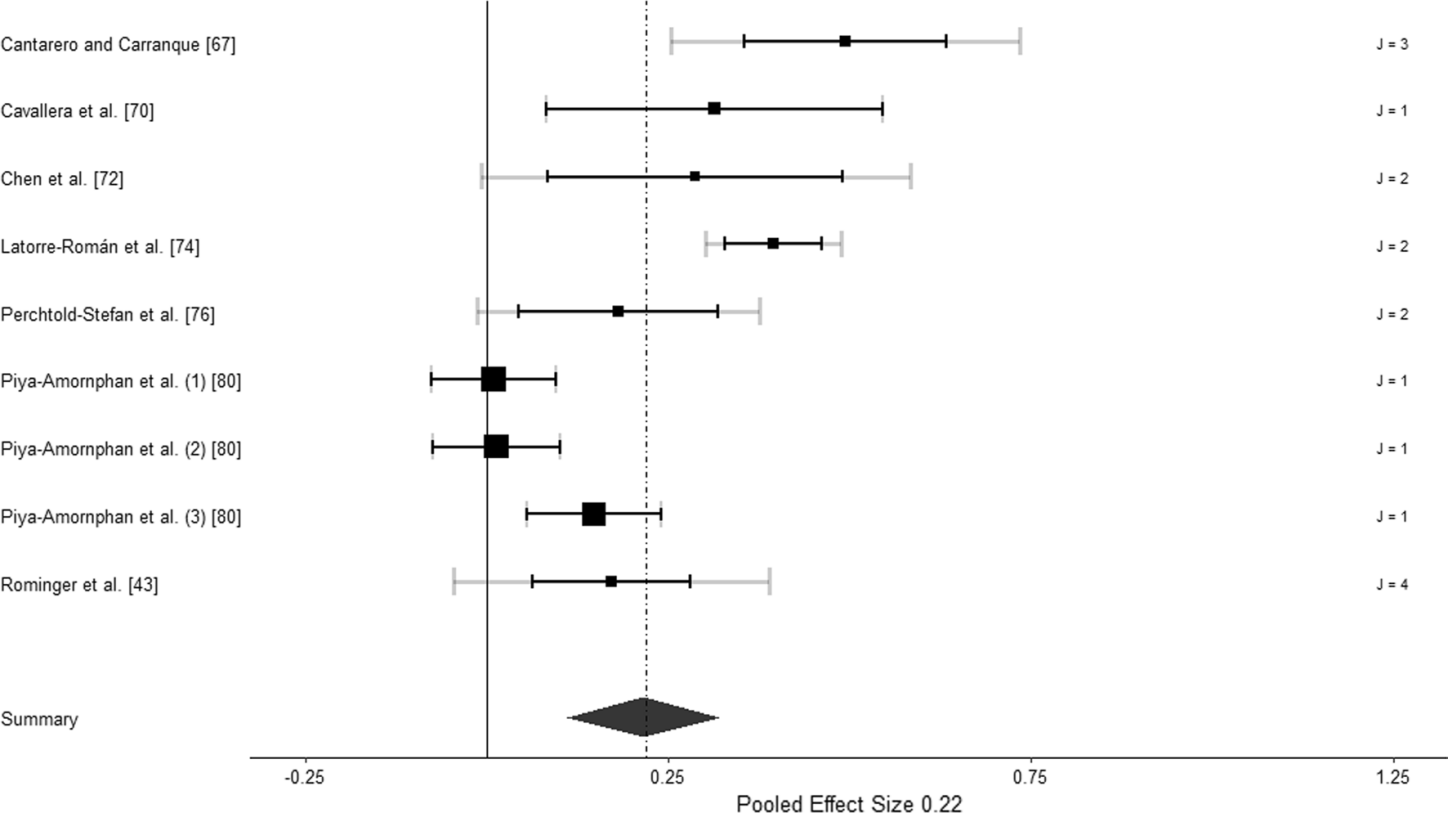
Forest plot of cross-sectional studies. J represents the number of outcomes. Black squares in the forest plot represent the meta-analytical study mean and the corresponding 95% confidence interval. Grey confidence intervals represent the sampling variance of observed effect sizes within each study and indicate the influence of the study sample mean on study precision. The thickness of the grey confidence intervals is proportional to the number of effect sizes within a study [66]. The numbers in parentheses designate the independent subsamples within a study

Seven studies were included for analysis, with a total of 17 effect sizes based on the data of 2073 participants. The aggregated effect was medium-sized, *r* = 0.22 (SE = 0.06, *p* = 0.002, 95% CI [0.10–0.34]). The estimated variance between studies (level 3) was *τ*^2^ = 0.025 (81.52%) and within studies (level 2) was *τ*^2^ = 0.000 (< 0.01%). 18.48% of the total variance could be attributed to the variance at level 1. Figure 2 shows a forest plot for all included effect sizes.

**Fig. 3:**
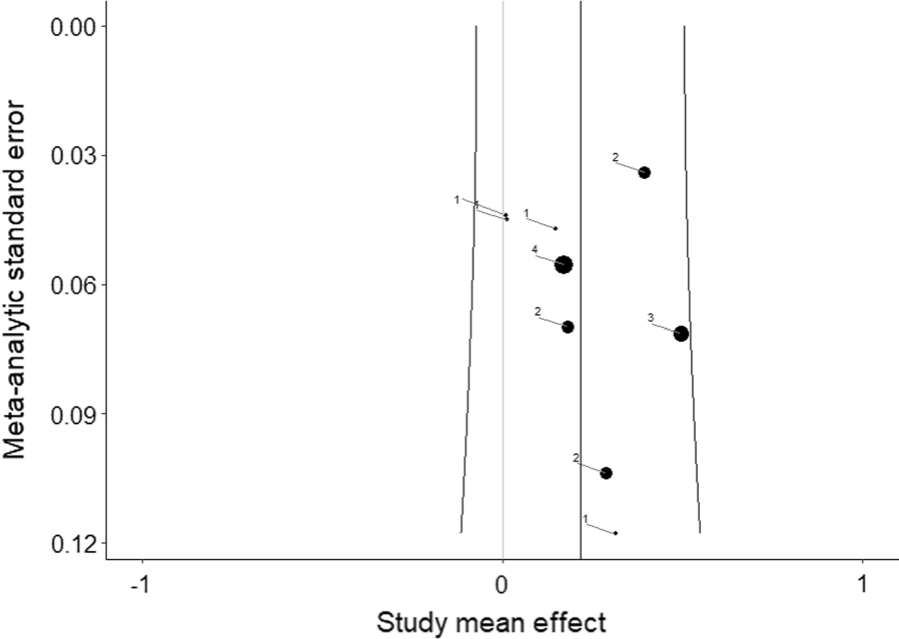
Funnel plot of cross-sectional studies. The size of the dots is proportional to the number of effect sizes included in the studies

Moderator analyses for indicators of creative ideation performance (*p* = 0.658), creativity domain (*p* = 0.727), population (*p* = 0.215), study quality (*p* = 0.586), gender (*p* = 0.271), and publication year (*p* = 0.060) were not significant. For details on moderator analysis, see Additional file 3. In line with Egger’s regression test (*p* = 0.228), the funnel plot did not indicate publication bias (Fig. 3).

### Intervention Studies

**Fig. 4:**
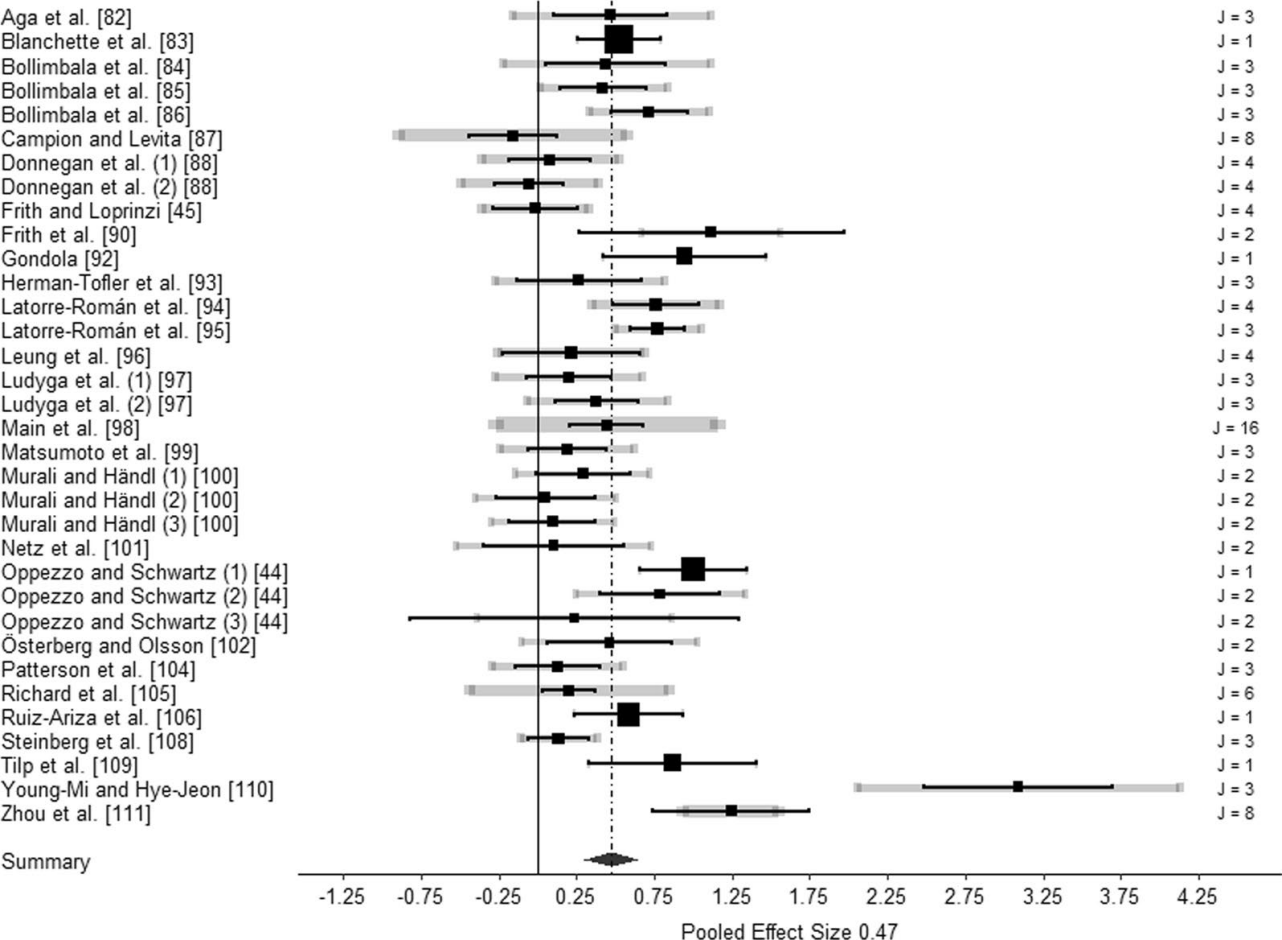
Forest plot of cross-sectional studies. J represents the number of outcomes. Black squares in the forest plot represent the meta-analytical study mean and the corresponding 95% confidence interval. Grey confidence intervals represent the sampling variance of observed effect sizes within each study and indicate the influence of the study sample mean on study precision. The thickness of the grey confidence intervals is proportional to the number of effect sizes within a study [66]. The numbers in parentheses designate the independent subsample within a study

Twenty-eight studies were included for analysis, with a total of 115 effect sizes and a total of 1624 participants. The aggregated effect size was medium, with Hedges’ *g* = 0.47 (SE = 0.09, *p* < 0.001, 95% CI [0.30–0.65]). The estimated variance components were at level 3 *τ*^2^ = 0.216 (62.65%) and at level 2 *τ*^2^ = 0.075 (21.84%). 15.51% of the total amount of variance could be attributed to the sampling variance at level 1. Figure 4 shows the forest plot of all effects.

**Fig. 5:**
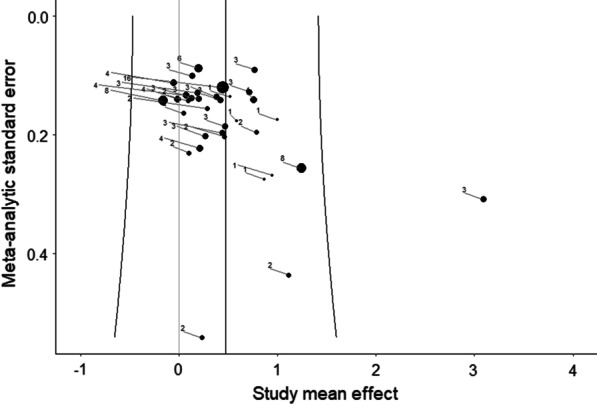
Funnel plot of intervention studies. The size of the dots is proportional to the number of effect sizes included in the studies

Moderator analyses for indicators of creative ideation performance (all *p* = 0.699), creativity domain (*p* = 0.549), population (*p* = 0.060), time of measurement (*p* = 0.198), and study design (*p* = 0.673) were not significant. Furthermore, the continuous moderator analysis for study quality (*p* = 0.720), gender (*p* = 0.083), and publication year (*p* = 0.840) were not significant. However, the significant moderator analysis for implementation of intervention (*p* = 0.020) showed that the mean effect of acute physical activity was lower (Hedges’ *g* = 0.37, *p* = 0.020) than the mean effect of chronic physical activity (Hedges’ *g* = 0.89, *p* < 0.001; details on moderator analysis see Additional file 3). In accordance with a significant Egger’s regression test (*p* = 0.009), the asymmetrical distribution of effects in the funnel plot was indicative of small-study effects, which could imply publication bias (see Fig. 5).

Robustness of the estimated effect was examined through outlier detection. The effects of studies were judged as outliers if the aggregated effect size exceeded ± 2 standard deviations. Due to this, we trimmed the available data and excluded five effect sizes (three from one study) from analysis. The nonsignificant Egger’s regression test of the trimmed dataset (*p* = 0.911) did not argue for an asymmetrical distribution of effects. The pooled effect size of the reduced dataset still indicated a significant Hedges’ *g* = 0.39 (SE = 0.06, *p* < 0.001, 95% CI [0.27–0.51]).

Further sensitivity analyses for the overall-effect were calculated for within-design studies applying *r* = 0.10 and *r* = 0.90 for the correlation between two time points to estimate the corresponding effect sizes. These analyses showed similar results for both correlations (*r* = 0.10: Hedges’ *g* = 0.47, *p* < 0.001; *r* = 0.90: Hedges’ *g* = 0.46, *p* < 0.001).

## Discussion

In this systematic quantitative review, which includes two meta-analyses, we analyzed the effect of physical activity on creative ideation performance. The cross-sectional meta-analysis indicated a positive association between habitual physical activity and creative ideation performance. The second meta-analysis showed that physical activity interventions enhance creative ideation performance. Moderator analysis indicated that even short periods of physical activity such as running or walking may boost creative potential. However, longer trainings showed stronger effects. Furthermore, physical activity did neither differentially affect verbal or figural creative ideation, nor the quantity (e.g., fluency) or quality (e.g., originality) of ideas. Neither population, gender, nor study quality moderated the observed effect sizes. The highest proportion of variance of effects was observed between studies at level 3, which could indicate high variability in methods for intervention as well as cross-sectional studies.

The included cross-sectional studies showed a medium effect of habitual physical activity on measures of creative ideation performance. This effect is in line with findings for cognitive functioning [39]. However, the pooled effect was based on seven methodologically heterogeneous studies. Five of these studies assessed habitual physical activity by self-report measures such as the Freiburger Questionnaire on Physical Activity (FQPA [79]; see Table 1) and only two studies applied behavioral measures of physical activity. Rominger et al. [43] used accelerometer sensors, which were worn for 5 days in everyday life, and Latorre Román and colleagues [74] applied a fitness test battery in a sample of 308 primary school students to measure physical activity. Although the meta-analysis on cross-sectional data indicated a medium-sized positive link between creative ideation performance and habitual physical activity, the causal direction of the effect in these studies remains unclear.

In order to investigate the causal effect of physical activity on creativity, the second part of this study examined intervention studies, which showed a medium-sized effect. In a recent narrative review, Frith et al. [15] concluded that the empirical rigor of available studies on the association between physical activity and creative ideation performance is quite diverse, which would consequently hamper the quality of meta-analyses. Basically, this criticism could also apply to the present meta-analysis. However, in this particular context it is important to consider that 16 further studies (46% of studies) have been published since Firth’s review. This indicates the increasing relevance of this research topic and the rapid accumulation of data. During the last decade, 86% of the seven cross-sectional studies and about 82% of the 28 included intervention studies have been published (see Tables 1 and 2). Furthermore, to address this critical issue statistically, we have added the continuous moderators of scientific quality to our multilevel analyses, which, however, did not show significant influences on the two pooled effect sizes.

While cross-sectional studies do not permit causal conclusions, their designs are much more robust [15]. This is particularly relevant because double-blinded intervention studies, which would control for potential placebo-effects, are not available in this field of research. This contrasts with studies investigating creativity-enhancing effects by means of substance intake, such as alcohol, cocaine, or caffeine [112–115], as well as brain stimulation studies [116–118]. In these experiments, the experimenter, the participants, and the originality raters can be blinded more easily. Furthermore, in these studies active control groups and control conditions only differ with respect to the dose of substance of interest.

All included studies in our meta-analyses used diverse methodological approaches, especially concerning the type of physical activity applied. Specifically, chronic physical activity intervention studies are less common, and most studies investigated the effects of single exercise sessions on creative ideation performance (75%; see Table 2). This might be one reason for the observed heterogeneity and high level of observed between-study variances of 63% for intervention studies. Nevertheless, most moderators of the present meta-analyses failed to show significance. This observation might have at least two potential explanations. First, these moderators have no effects. The null finding for *indicator of creativity domain* and *indicators of creative ideation performance* is in line with a meta-analysis reporting similar enhancing effects of chronic physical activity for memory, attention, and executive functions [119]. A second explanation might be a lack of statistical power to detect such moderators, specifically when looking at meta-analysis on more basic cognitive functions. A recent meta-analysis of Ludyga et al. [119] analyzed 80 studies, and the review of reviews of Biddle et al. [6] even reported the aggregated information of 392 studies. In this context, it should be noted that gender showed a trend for significance to moderate the pooled effect of the present meta-analysis. Physical activity interventions led to lower enhancement as the percentage of women increased. This finding is well in accordance with Ludyga et al. [119], who showed a generally small improvement in cognitive function after chronic interventions, which was less pronounced in women. In addition, due to the high heterogeneity of approaches to describe and report exercise interventions (e.g., intensity), the study details are not sufficient to investigate potentially relevant moderators, such as type of exercise and exercise intensity. This warrants further empirical studies with high methodological rigor [15]. Particularly, cross-sectional studies have the great potential to complement the findings of randomized and well-controlled intervention studies [39, 72]. This would be important to improve the quality and quantity of available data, which would in turn allow to investigate potential moderating variables in greater detail. The increase in available studies should also ameliorate the observed publication bias (for intervention studies), which did not strongly impact the reported study results. After adjustment for publication bias (via trimming of high effects), the effect size remained quite similar. Notwithstanding these restrictions, the present meta-analyses revealed a reliable and robust effect of acute, chronic, and habitual physical activity on creative ideation performance [2].

The meta-analysis of intervention studies indicated an effect of physical activity on creative ideation performance, which was more pronounced for chronic in contrast to acute (single bouts of) physical activity [36]. This finding is in line with Etnier et al. [2], who reported stronger effects for chronic interventions on cognition (but see [42] for children). Although differential effects on more basic cognition are well known, the relevant mechanisms for chronic and acute interventions are still poorly understood.

### Presumed Psychological Mechanisms

The findings of the present meta-analyses are in line with the general assumption that physical activity offers a change, that is the transition from one state to the other, which could enhance the quality and the quantity of ideas by preparing people to deal with these changes [96, 98]. Main et al. [98] showed in three experiments that changes in physical activity enhanced creative thinking, while inactivity or repetitive physical activity lowered it. However, this line of argumentation is strongly restricted to acute effects of physical activity and does not provide explanations for the stronger chronic effects of physical activity on creative ideation performance. A second suggested mechanism why physical activity could boost creative ideation performance besides gearing for change is that acute as well as chronic physical activity is associated with higher positive affect and reduced negative affect and stress [120–123]. Based on the broaden and build theory, which assumes that positive affect coincides with more original ideas via cognitive flexibility [65, 124], the physical activity-associated affective shift was suggested to increase creative ideation performance [43, 87, 125]. Although this hypothesis has been investigated with cross-sectional and intervention studies, to the best of our knowledge, no study to date has confirmed the implied mediating role of positive affect on creative ideation outcome [43, 82, 98, 108]. However, as outlined by Biddle et al. [6], the effects of physical activity on stress (negative affect) and positive affect in ecologically valid settings could be a fruitful area for future research [43, 126]. The collection of extensive longitudinal data might provide a more detailed picture of the mechanisms of physical activity on affect and creative ideation performance [126]. Besides these psychological mechanisms, physiological mechanisms may also explain the enhancing effect of physical activity on creative cognition. If sports, exercise, and fitness training stimulate creative cognition via improvements in cognitive functioning, such as executive control, attention, and memory processes [7, 119], similar physiological mechanisms might be valid for both domains.

### Presumed Physiological Mechanisms

Changes in the brain-derived neurotrophic factor (BDNF), structural changes in brain areas, functional connectivity changes, as well as changes in blood flow and neurotransmitters constitute powerful physiological mechanisms tightly linked to physical activity [2, 119, 127–130]. Although acute physical activity can induce changes in BDNF, (cerebral) blood flow, and the release of neurotransmitters dopamine and norepinephrine, structural and functional physiological changes are more strongly related to chronic interventions [5, 7, 38]. Accordingly, a two-week running intervention induced structural brain changes associated with improvements in participants’ affective functions. Fink et al. [131] further showed that (short) chronic physical activity interventions might have the potential to modulate psychological functions via changes in brain structure [39, 130]. Since positive affect is associated with creative ideation performance [43], this study might hence provide one potential line of reasoning of how creative ideation could be modulated by chronic physical activity-induced brain changes. A meta-analysis on fMRI activation data indicated that physical exercise leads to activation changes in the precuneus, frontoparietal networks, and default mode networks [132], which are associated with creative ideation performance [25, 26, 133–136]. This is in accordance with reviews concluding that physical activity has the potential to improve the structural plasticity and function of the brain throughout the life span, specifically in neurological and psychiatric patients [5, 7, 39, 137].

The neuro-protective role of physical activity was coined *brain health* and could constitute an important target of future creativity research [5, 7, 39, 41], where creative ideation performance may serve as a meaningful behavioral indicator of a flexible, original, innovative, and healthy brain in children as well as in adults. Importantly and critically, however, research on boosting creative performance by means of physical activity needs further rigorous and well-powered investigations in randomized controlled trials and with proper control groups involving neurophysiological methods (see [39], for cognitive functions). Specifically, the dose–response relationship (i.e., effect of duration, repetition, and intensity) between physical activity and creative ideation performance increases seems to be an important target for future research in order to further confirm causality [6, 9, 34, 53, 119].

## Conclusion

Creative cognition is relevant for team sports [138], and physical activity and sports, in turn, can boost creative ideation performance. The present meta-analysis highlights this synergistic effect [39] by suggesting an impact of physical activity on creative idea generation. The observed enhancing medium-sized effect is in line with the current state of knowledge assuming a boost of basic cognitive functions, such as executive control, attention, and memory due to physical activity [2, 7, 119]. Furthermore, the present study expands this evidence to the field of creativity research and suggests that physical activity could increase the complex and dynamic interplay of cognitive functions, ultimately facilitating creative ideation. The present findings encourage further investigations in representative samples to learn more about the mechanisms of creativity enhancement via physical activity, which is a growing field of interest relevant for health, sport, and sports medicine [72, 82, 139]. In the future, rigorous cross-sectional and experimental intervention research could help to further develop public health recommendations for physical activity as an important lifestyle factor [39].

## Supplementary Information

**Additional file 1**. Search string.

**Additional file 2**. Study quality assessment protocol.

**Additional file 3**. Results of moderator analyses.

## Data Availability

All data are available on request.

## Code availability

*R* script used in this study is available on request.
