# Supplementary material for: Acute and Chronic Physical Activity Increases Creative Ideation Performance: A Systematic Review and Multilevel Meta-analysis
Source: Sports Med Open. 2022 May 6;8:62. doi: 10.1186/s40798-022-00444-9 (PMC9076802; doi:10.1186/s40798-022-00444-9)
Supplement: Supplementary file 1 — Additional file 1. Search string. [file 40798_2022_444_MOESM1_ESM.docx]

**Search string**

**Scopus**:

("physical activity" OR "bodily movement" OR "aerobic exercise" OR "exercise program" OR danc* OR "physical fitness" OR walk* OR running ) AND ( creativ* OR "creative thinking" OR "divergent thinking" OR "creative potential" OR "open problem solving" OR "creative ideation" OR originality OR fluency )

**Psyarxiv:**

Creativity AND Movement

**Google Scholar (first 300 pages)**

("physical activity" OR "bodily movement" OR "aerobic exercise" OR "exercise program" OR "physical fitness") AND (creativity OR “creative thinking” OR “creative potential” OR “open problem solving” OR “creative ideation” OR fluency)

**Zenodo**:

("physical activity" OR "bodily movement" OR "aerobic exercise" OR "exercise program" OR dancing OR "physical fitness" OR walking OR running) AND (creativity OR “creative thinking” OR “divergent thinking” OR “creative potential” OR “open problem solving” OR “creative ideation” OR originality OR fluency)

**PsycInfo:**

("physical activity" or "bodily movement" or "aerobic exercise" or "exercise program" or dancing or "physical fitness" or walking or running) and (creativity or "creative thinking" or "divergent thinking" or "creative potential" or "open problem solving" or "creative ideation" or originality or fluency)

**PubMed:**

("physical activity" OR "bodily movement" OR "aerobic exercise" OR "exercise program" OR danc* OR "physical fitness" OR walk* OR running) AND (creativ* OR "creative thinking" OR "divergent thinking" OR "creative potential" OR "problem solving" OR "creative ideation" OR originality OR fluency)

**Web of Science:**

("physical activity" OR "bodily movement" OR "aerobic exercise" OR "exercise program" OR dancing OR "physical fitness" OR walking OR running) AND (creativity OR “creative thinking” OR “divergent thinking” OR “creative potential” OR “open problem solving” OR “creative ideation” OR originality OR fluency)

**Cinahl**:

("physical activity" OR "bodily movement" OR "aerobic exercise" OR "exercise program" OR dancing OR "physical fitness" OR walking OR running) AND (creativity OR “creative thinking” OR “divergent thinking” OR “creative potential” OR “open problem solving” OR “creative ideation” OR originality OR fluency)

**ProQuest:**

("physical activity" OR "bodily movement" OR "aerobic exercise" OR "exercise program" OR dancing OR "physical fitness" OR walking OR running) AND (creativity OR “creative thinking” OR “divergent thinking” OR “creative potential” OR “open problem solving” OR “creative ideation” OR originality OR fluency)
