# Supplementary material for: Acute and Chronic Physical Activity Increases Creative Ideation Performance: A Systematic Review and Multilevel Meta-analysis
Source: Sports Med Open. 2022 May 6;8:62. doi: 10.1186/s40798-022-00444-9 (PMC9076802; doi:10.1186/s40798-022-00444-9)
Supplement: Supplementary file 2 — Additional file 2. Study quality assessment protocol. [file 40798_2022_444_MOESM2_ESM.docx]

Table S1. Study quality assessment protocol for intervention studies.

| Item | Longitudinal study | Rating |
| --- | --- | --- |
| (1) | Was the physical activity manipulation controlled (e.g., completed in a laboratory setting, standardized by duration and intensity, and for interaction with other participants if administered in a group context)? * | Yes (1)/No (0) |
| (2) | Was there evidence of validity for the creativity measure(s) utilized? | Yes (1)/No (0) |
| (3) | Was there evidence of reliability for the creativity measure(s) utilized? (e.g., reporting of Cronbach’s Alpha of reported data) | Yes (1)/No (0) |
| (4) | Were creativity scoring and evaluation procedures robust to bias (e.g., blinded scoring completed by multiple researchers, provision of strong interrater reliability)? | Yes (1)/No (0) |
| (5) | Was the order of creativity assessments randomized to ensure resistance to temporal artifacts or learning effects? | Yes (1)/No (0) |
| (6) | Did the intervention use a non-exercise control group or condition? * | Yes (1)/No (0) |
| (7) | Where all examined effects reported? | Yes (1)/No (0) |
| (8) | Was the study a randomized control trial? * | Yes (1)/No (0) |

* For cross-sectional study: (1) Was the physical activity assessed by behavioral measure? The questions (6) and (8) are not applicable.
