# Supplementary material for: Acute and Chronic Physical Activity Increases Creative Ideation Performance: A Systematic Review and Multilevel Meta-analysis
Source: Sports Med Open. 2022 May 6;8:62. doi: 10.1186/s40798-022-00444-9 (PMC9076802; doi:10.1186/s40798-022-00444-9)
Supplement: Supplementary file 3 — Additional file 3. Results of moderator analyses. [file 40798_2022_444_MOESM3_ESM.docx]

**Cross-sectional studies - Moderators**

| **Categorical moderators** |  |  |
| --- | --- | --- |
|  | *r* | *p*-value |
| *Indicators of creative ideation* |  |  |
| Fluency [reference category] | 0.266 | 0.658 |
| Flexibility | 0.363 |  |
| Originality | 0.314 |  |
| Elaboration | 0.253 |  |
| Composite score | 0.144 |  |
| *Creativity domain* |  |  |
| Verbal | 0.192 | 0.727 |
| Figural [reference category] | 0.210 |  |
| *Population* |  |  |
| Adults | 0.143 | 0.215 |
| Children [reference category] | 0.287 |  |
| **Continuous moderators** |  |  |
|  | *β* | *p*-value |
| Study quality | 0.049 | 0.586 |
| Gender (percentage of female participants) | -0.009 | 0.271 |
| Publication year | -0.037 | 0.060 |

**Intervention studies - Moderators**

| **Categorical moderators** |  |  |
| --- | --- | --- |
|  | Hedges’ g | *p*-value |
| *Indicators of creative ideation* |  |  |
| Fluency | 0.447 | 0.699 |
| Flexibility | 0.401 |  |
| Originality | 0.551 |  |
| Elaboration | 0.471 |  |
| Composite score | 0.520 |  |
| *Creativity domain* |  |  |
| Verbal | 0.456 | 0.549 |
| Figural | 0.539 |  |
| *Population* |  |  |
| Adults | 0.734 | 0.060 |
| Children | 0.365 |  |
| *Time of measurement* |  |  |
| During | 0.627 | 0.198 |
| After [reference category] | 0.428 |  |
| *Study design* |  |  |
| Between | 0.525 | 0.673 |
| Within [reference category] | 0.445 |  |
| *Implementation of intervention* |  |  |
| Acute [reference category] | 0.374 | 0.020 |
| Chronic | 0.891 |  |
| **Continuous moderators** |  |  |
|  | *β* | *p*-value |
| Study quality | -0.022 | 0.720 |
| Gender (percentage of female participants) | -0.012 | 0.083 |
| Publication year | -0.002 | 0.840 |
